# Study roadmap for high-throughput development of easy to use and affordable biomarkers as diagnostics for tropical diseases: a focus on malaria and schistosomiasis

**DOI:** 10.1186/s40249-017-0344-9

**Published:** 2017-10-02

**Authors:** Kokouvi Kassegne, Ting Zhang, Shen-Bo Chen, Bin Xu, Zhi-Sheng Dang, Wang-Ping Deng, Eniola Michael Abe, Hai-Mo Shen, Wei Hu, Takele Geressu Guyo, Solomon Nwaka, Jun-Hu Chen, Xiao-Nong Zhou

**Affiliations:** 1 National Institute of Parasitic Diseases (NIPD), Chinese Centre for Disease Control and Prevention, Shanghai, 200025 People’s Republic of China; 2 WHO Collaborating Centre for Tropical Diseases, National Centre for International Research on Tropical Diseases, Key Laboratory of Parasite and Vector Biology of the Chinese Ministry of Health, Shanghai, 200025 People’s Republic of China; 3 Department of Microbiology and Microbial Engineering, School of Life Science, Fudan University, Shanghai, 200433 People’s Republic of China; 4 African Network for Drugs & Diagnostics Innovation (ANDI), Addis Ababa, Ethiopia

**Keywords:** Malaria, Schistosomiasis, Neglected tropical diseases, Immunoproteomics, Protein microarray, Serological biomarker, Diagnosis

## Abstract

**Background:**

Interventions are currently being used against ‘infectious diseases of poverty’, which remain highly debilitating and deadly in most endemic countries, especially malaria, schistosomiasis, echinococcosis and African sleeping sickness. However, major limitations of current ‘traditional’ methods for diagnosis are neither simple nor convenient for population surveillance, and showed low sensitivity and specificity. Access to novel technologies for the development of adequate and reliable tools are expressly needed. A collaborative project between African Network for Drugs and Diagnostics Innovation and partner institutions in Africa and China aims to screen suitable serological biomarkers for diagnostic pipelines against these ‘diseases of the poor’.

**Methods:**

Parasite-specific exposed versus unexposed individuals were screened and sera or urine/stools were collected through case-control studies in China and African countries. Target genes/open reading frames were selected, then will be cloned and cell-free expressed, quantified and immuno-detected. Target antigens/epitopes will be probed and screened with sera from exposed or unexposed individuals using a high-throughput antigen screening platform as the study progresses. The specificity and sensitivity of highly immunoreactive biomarkers will be evaluated as well, using enzyme-linked immunosorbent assays or dipsticks.

**Discussion:**

This roadmap explicitly unfolds the integrated operating procedures with focus on malaria and schistosomiasis, for the identification of suitable biomarkers that will aid the prioritization of diagnostics for population use. However, there is need to further validate any new diagnostic through comparison with standard methods in field deployable tests for each region. Our expectations for the future are to seek regulatory approval and promote the use of diagnostics in endemic areas.

**Electronic supplementary material:**

The online version of this article (doi:10.1186/s40249-017-0344-9) contains supplementary material, which is available to authorized users.

## Multilingual abstract

Please see Additional file 1 for translations of the abstract into the five official working languages of the United Nations.

## Background

Malaria, schistosomiasis, echinococcosis and African sleeping sickness are among the diseases causing serious morbidity and mortality in Sub-Saharan Africa [1–3]. The World Health Organization (WHO) reported 212 million new cases of malaria infection resulting in 429,000 deaths globally in 2016, and over 90% of the deaths were in Africa. The majority (62%) of the cases occurred in children under 15 years old and about 125 million pregnant women are at risk of infection every year in sub-Saharan Africa [4]. Schistosomiasis is prevalent in about 50 African countries causing more than 200,000 deaths per year in sub-Saharan Africa, while echinococcosis and African sleeping sickness are both endemic in more than 30 countries, and together, are responsible for about 12,000 deaths per year [3, 5]. In most endemic countries, these infections disproportionately affect the poor and disadvantage people who have limited access to health facilities. They are not just diseases commonly associated with poverty, but they are also a cause of poverty and a major hindrance to economic development. Poverty can increase the risk of these diseases since those in poverty do not have the financial capacities for prevention or treatment. In its entirety, the economic impact of malaria has been estimated to cost Africa US$12 billion every year. The economic impact includes costs of health care, working days lost due to sickness, days lost in education, decreased productivity due to neurological disabilities from cerebral malaria in African children, and loss of investment and tourism [6]. Furthermore, current estimates suggest that echinococcosis results in the loss of 871,000 disability-adjusted life years (DALYs) annually, and associated annual costs are estimated to be US$ 3 billion for treating cases [5].

Efforts have been made using a vast repertoire of interventions against these diseases, including; preventive chemotherapy, case-detection and case management, vector and intermediate host control, veterinary public health at the human – animal interface, and provision of safe water, sanitation and hygiene [7–9]. These are being strengthened by other approaches such as technical guidance to increase preventive measures, diagnostics, treatment and disease surveillance [10–13]. In endemic countries, diagnosis methods for human parasites are mainly focused on parasitological or clinical examinations. For instance, microscopic examination of blood smears is used for malaria *Plasmodium* and Kato-Katz stool examination for *Schistosoma* parasites. Major limitations of these current ‘traditional’ diagnosis methods are neither simple nor convenient for population surveillance, and showed low sensitivity and specificity [14, 15]. Such strategies alone without harnessing innovation and expanding research make the goal of combating these infections arduous, especially in low- and mid-income countries [16, 17]. This implies that the lack of specific assays for population use or field diagnosis constrains researchers and stakeholders to develop antigen/antibody-based diagnostics. Thus, post-genomic research of human parasites has boosted the understanding of the detailed aspects of antigen-specific human immune responses for diagnosis purposes. The so called ‘traditional’ proteomic approaches based on; expression cloning, 2-dimensional (2-D) liquid chromatography elution and mass spectrometry, reverse immunogenetics, and later, conventional techniques in immunology such as 2-D gel electrophoresis, western blotting and enzyme-linked immunosorbent assay (ELISA), have given insights on antigen-antibody reactivity. They have shown that protein targets are immuno-reactive against infected individual antibodies and have been useful to identify candidate antigens and also helpful in high-throughput (HTP) protein profiling [18]. However, these approaches have not been able to evaluate HTP antibody profiling properly, because they are unsuitable considering the complexity of the humoral immune responses profiling, and also inadequate to efficiently identify large amount of promising candidate targets [18–21]. Therefore, it is imperative to strengthen and implement novel technologies for the development of health tools for field diagnosis or specific assays for population use to effectively control and eliminate the ‘infectious diseases of poverty’ [14, 15, 22]. HTP protein microarrays have been proposed and have given the opportunity to explore and analyse natural humoral immune responses, in identifying potential target antigens for diagnosis and vaccine pipelines [18]. In other words, the concept of immunoproteomic HTP assays has been set-up to change the manner in which ‘traditional’ immunological researches were practised in order to identify appropriate biomarkers for diagnostics or vaccines.

An award from the World Health Assembly (WHA), the World Health Organization (WHO) Demonstration Project titled “Development for Easy to Use and Affordable Biomarkers as Diagnostics for Types II and III Diseases” is a collaborative project between African Network for Drugs and Diagnostics Innovation (ANDI) and partner institutions in Africa and China. This project aims to leverage well-established HTP antigen screening platform, known as an omics-based technology developed by the Chinese Network for Drugs and Diagnostics Innovation (Chinese NDI) research group, to discover biomarkers and process them for diagnostics development. Hence, the Chinese NDI organized one month training workshop for capacity building and knowledge transfer to young African scientists from participating institutions and fellows from collaborating institutions in September 2016. This project targets to screen biomarkers for malaria, schistosomiasis, echinococcosis and African trypanosomiasis at genome-wide scale of approximately 10,000 proteins. However, in the first year of a 5-year period project, only 700–800 proteins were targeted. Antigens/epitopes that will be associated with these infections will be identified as well, and diagnostic kits will be further translated into field for population use. This is one of the important steps taken to using an immunomics-based technology to develop such sensitive, specific and affordable new diagnostic tools against these infections.

Herein, we stress the protocol study, especially with a focus on malaria and schistosomiasis, describing the processes involved in the identification/development of serological biomarkers for diagnostic pipelines. Updates on existing genes, proteins and omic databases have been made to leverage this study.

## Methods/Design

The **s**pecific aims of the study are as follows: 1) Develop protein microarrays containing selected antigens for each disease; 2) Probe well-characterized infected human sera from China and African countries and identify serodiagnostic antigens; 3) Develop, evaluate, validate and optimize field deployable tests for each agent applicable to each region; and 4) Seek regulatory approval and promote the use of products in endemic area.

**Fig. 1:**
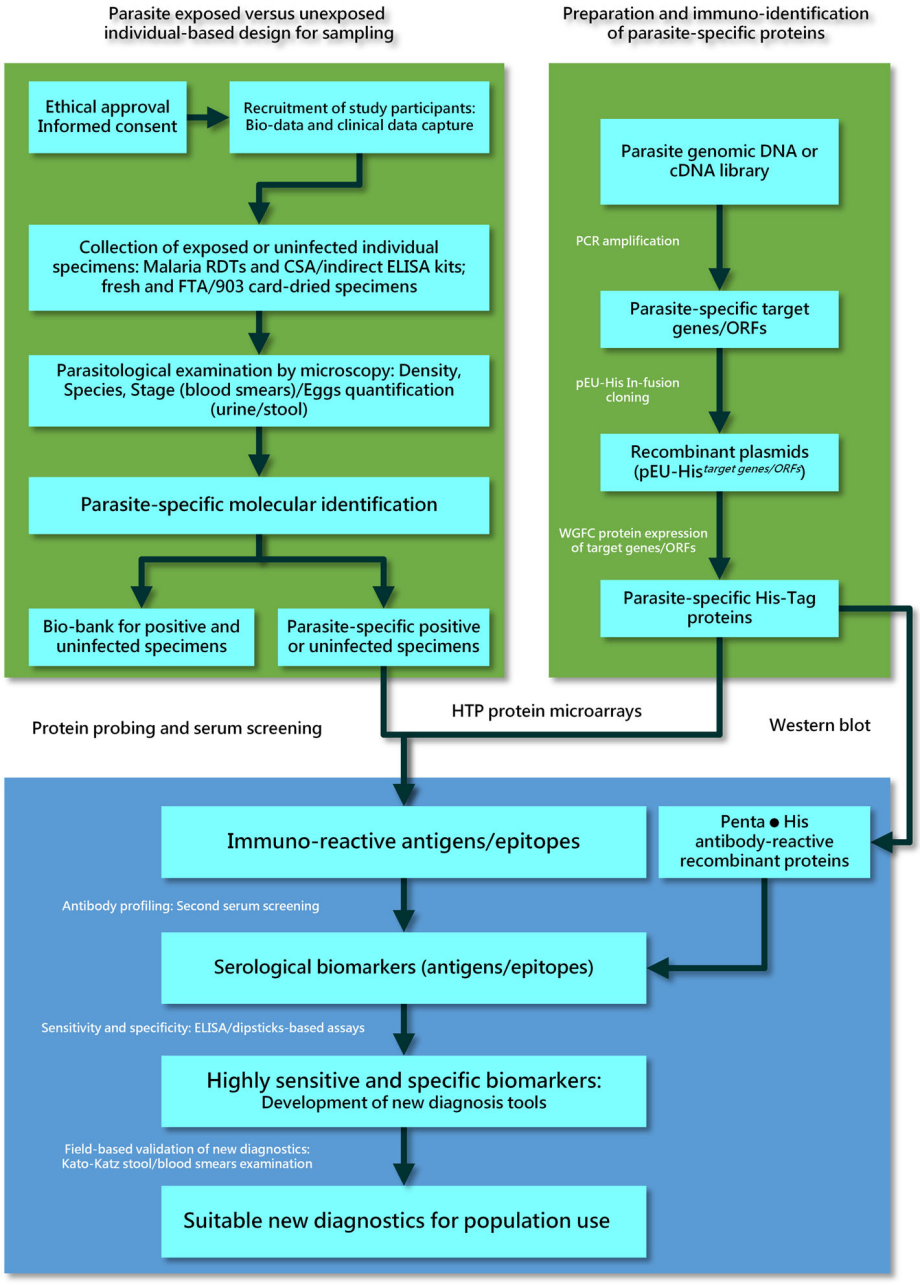
Flow chart for the processes involved in the identification of human parasite biomarkers for diagnostic development in the context of the WHA-WHO Demonstration Project

The scientific study roadmap for this project includes; characterization and collection of parasite-infected or uninfected individuals’ serum and urine/stool samples from China and African countries, development of HTP protein microarrays of selected targets through protein probing and subsequent sera screening, culminating to the identification/discovery of serodiagnostic antigens/epitopes. It should be noted that updates of omic databases and all the laboratory work will be performed in China. Figure 1 shows an overview of the flow chart for the processes involved and discussed below.

### Update of existing genes, proteins and omic databases

The study will contribute greatly to knowledge as it aims to screen and update suitable biomarkers for *Plasmodium* and *Schistosoma* diagnostics. Malaria and schistosomiasis with strains including *P. falciparum*, *S. haematobium* and *S. mansoni* respectively are predominant in Africa [1, 23–28], whereas *S. japonicum* and *P. vivax* are prevalent in China [29–33]. Updates of *Plasmodium* and *Schistosoma* genes, proteins and omic databases have been achieved by the Chinese NDI, though, some have been identified in our previous studies [19–21, 34]. These updates include; molecular and immunological characterization of targets, and development of protein microarrays with individual infected serum samples.

### Collaborative institutions, study sites and repartition of sampling

National Institute of Parasitic Diseases, Chinese Centre for Disease Control and Prevention, and Fudan University (China), Theodor Bilharz Research Institute (Egypt), University of Health and Allied Sciences (Ghana), Kenya Medical Research Institute (Kenya) and University of Lagos (Nigeria) are the collaborative institutions involved in this project. The study sites are located in China or African countries where malaria and schistosomiasis are endemic and specimens have been collected. The distribution of parasite-infected individual specimens is shown in Table 1.

**Table 1:** Repartition of study sites and sample collection for malaria and schistosomiasis specimens

		China	Ghana	Kenya	Nigeria
		(Number of parasite-infected individual specimens)			
Parasite-infected individual specimens	*P. falciparum*	150^a^	150^c^	150^d^	150^f,g^
	*P. vivax*/other species	150^a^	50^c^	50^d^	50^f,g^
	*S. japonicum*	150^b^	Not required	Not required	Not required
	*S. haematobium*	Not required	150^c^	150^d^	150^f,g^
	*S. mansoni*	Not required	150^c^	150^e^	150^g^

^a^Tengchong County, Yunnan Province, China-Myanmar endemic border areas [32]

^b^Guichi and Tongling Counties, Anhui Province, Lake Region, Mid-East [33]

^c^Ho City, Ho Municipal District, Volta Region [27, 28]

^d^Msambweni, Kwale County, Coast Region [23, 25]

^e^Kangundo, Machakos County, Eastern Region [23]

^f^Okorodu/Badagry Districts, Lagos State [24, 26, 45]

^g^Wamako/Wurno Districts, Sokoto State [24, 26, 45]

### Ethical approval

The project’s sampling method was approved by the Ethics Review Committee of the National Institute of Parasitic Diseases, Chinese Centre for Disease Control and Prevention. The study protocol, potential risks and potential benefits were explained to the participants. Only subjects under 65 years old were incorporated into the study. Informed consent was sought from the participants and written informed consents were obtained from the participants or their parents or guardians before sampling was done.

### Sampling: Case-control design

This has been revealed to be the key section in the antigen screening processes. This section is important because the source of antisera that will be used to profile serological responses and provide knowledge on pathogen-host interactions may give a bias understanding of the host immune responses [18]. Case-control design also known as the comparison of parasite-exposed versus unexposed individuals was considered among the three study designs that are used for the acquisition of antisera to identify antigen for diagnostic development [18]. Thus, the participants from China and African countries were screened. Exposed or clinically suspected and unexposed subjects were targeted for sampling, and their bio-data and clinical data were obtained. Clinical and parasitological information were captured using a standard questionnaire. Individuals with malaria and schistosomiasis co-infection were also of interest. Infected samples were collected from endemic areas, but uninfected samples were collected from areas that are non-endemic or with low endemicity.

Blood and urine/stool samples were collected from participants. Blood samples were immuno-identified using malaria Rapid Diagnostic Tests (RDTs, *CareSart*) and circulating *Schistosoma* antigen (CSA)/indirect enzyme-linked immunosorbent assay (ELISA) detection kits (*EASE-Medtrend*) respectively. Specimens that were RDTs and CSA/indirect ELISA positive were followed by microscopy for parasitological examinations. These include; blood smears for the density, species and stages of *Plasmodium* specimens*,* and urine/stools analysis for *Schistosoma* egg detection and load quantification*.* In this study, clinical malaria is defined as fever ≥37.5 °C and asexual parasitemia ≥2500 μl of blood [35], whereas clinical schistosomiasis is characterized as bloody stool/urine ≥100 eggs per gram of faeces or ≥50 eggs per 10 ml of urine [36]. Molecular screening by Polymerase Chain Reaction (PCR) amplification was required for unexposed specimens that were  RDTs and CSA/indirect ELISA negative to validate whether they were truly negative and may be fully use as controls (Fig. 1). Bio-banks for both infected and uninfected specimens were constructed in China and collaborative institutions in African countries.

### Selection of parasite target genes/open reading frames (ORFs)

For the first year of a 5-year period project, only 700–800 proteins were targeted. *P. falciparum* and *P. vivax* genes coding for merozoite surface proteins (MSPs) or erythrocyte-infected variant surface proteins (VSPs), with *Schistosoma japonicum*, *S. haematobium* and *S. mansoni* tegument or secretory proteins were selected [37–40]. More soluble and secretory proteins of these infections will be included, as well as for echinococcosis and African sleeping sickness as the study progress further. As advised, the constructs for gene expression do not contain any nucleotide sequence for signal peptide [41] and glycosylphosphatidylinositol (GPI) anchor [42].

### Preparation of linearized vector and PCR amplification of selected genes/ORFs

A vector, called pEU-E01-His-N2 (*CellFree Sciences*) was used for HTP cloning. The vector was linearized by double digestion with restriction enzymes *Xho* I and *Bam*H I (*Takara*) and purified according to manufacturer’s instruction to allow efficient joining of the PCR products with the vector.

Parasite-specific target genes/ORFs were PCR amplified using cDNA for nucleotidic sequences containing intron(s), or genomic DNA for those without intron. PCR products were generated in a way that they have fifteen bases which are homologous of the vector at their linear ends for coupling of the cloning vector with the targets. Thus, each gene-specific primer was converted into a specific primer by extension at the 5′ end as follows: 5′-GGG CGG ATA TCT CGA G-3′ and 5′-GCG GTA CCC GGG ATC CTT A-3′ for forward and reverse primers, respectively [20, 21].

### In-fusion cloning of target genes/ORFs

HTP In-fusion methods were used to efficiently clone amplified targets as reported previously [19–21, 34]. This technology allows for a perfect coupling of linearized vector and amplified DNA inserts in a ligation-independent – unidirectional cloning reaction for further protein expression with accurate engineered His-tags. The detailed process was reported in previous studies [20, 21]. PCR products were treated with cloning enhancer for a while, and ligated with a pEU-E01-His-N2 linearized vector in an In-fusion reaction. Recombinant plasmids (pEU-E01-His-N2^*target gene/ORF*^) were transformed into *Escherichia coli* DH5α (*Takara*) competent cells, and then plated onto ampicillin-based Luria-Bertani (LB) agar for overnight incubation.

### Wheat germ cell-free (WGCF) expression of target genes/ORFs

Positive clones are required for plasmid preparation and in vitro expression. WGCF (*CellFree Sciences*) expression is a unique HTP protein expression system that is qualified to overcome the major bottleneck in the research for antigen discovery of human parasite infections [43, 44]. In other words, the system has the capacity to efficiently synthesize large amount of proteins from genes which contain very high A/T sequences with high speed and precision [19, 20, 34]. Highly purified recombinant plasmid DNA sequences will be HTP transcripted and translated to crude proteins in in vitro bilayer reactions with any purification requirement as stated by the manufacturer.

### Quantitation and immuno-detection of recombinant proteins

The detection-quantitation and expression level of WGFC crudely expressed proteins will be determined using bicinchoninic acid (BCA) protein assay and sodium dodecyl sulfate polyacrylamide gel electrophoresis (SDS-PAGE) with Coomassie Brilliant Blue R-250 PAGE staining method. Protein concentrations are determined with reference to standards of a common protein in BCA protein assay. A series of dilutions of known concentration will be prepared from the protein and assayed alongside the unknowns; this allows for the determination of each unknown concentration from the standard curve. Furthermore, crude recombinant N-terminal His-tag fusion proteins will be immuno-detected using Western blot analysis. The total fraction of each sample will be separated by SDS-PAGE under reducing conditions, and then transferred onto polyvinyl difluoride (PVDF) membranes (*Millipore*). Penta ● His antibody (*Qiagen*) and horseradish peroxidase (HRP)-conjugated goat anti-mouse immunoglobulin G (*Pierce*) will be used to detect His-tagged recombinant proteins.

### HTP protein microarrays: Protein probing and serum screening

Protein probing onto a microarray-specific slide and serum screening constitute the main components of this innovative technology. A well-type amine array as described by Chen et al. [21] will help identify serological biomarkers of human parasites as the project progresses. First, crude WGCF recombinant proteins, as well as positive and negative controls will be spotted as probes in duplicate on a microarray-specific slide and incubated. Then, serum samples from parasite-specific exposed versus unexposed individuals will be used to screen the probes. Bound antibodies will be visualized using Alexa Fluor 546 goat anti-human IgG (*Invitrogen*), scanned in a fluorescent microarray scanner (*CapitalBio*), and fixed circle approach will be used to quantify the arrays.

Furthermore, screening of the highly immuno-reactive antigens/epitopes will be performed to profile their immune responses using also sera from infected individuals and healthy subjects.

### Data analysis: Bio-informatic approaches to immunology

Positive or high response of arrayed proteins against infected versus uninfected sera is defined as a relative ratio of signal intensity (SI) > 2.0 compared to the negative control SI. Two-tailed unpaired (GraphPad Prism software) and Benjamini-Hochberg (MULTTEST procedure of SAS/STAT) methods will be used to evaluate the immuno-reactivity of antigens/epitopes that will be identified through the correlate between probes and antibody reactivity, and to correct false discovery rate, respectively. Also, TIGR multi-array experiment viewer (MeV) will be used to draw and analyse the heatmap of humoral responses [20, 21].

### Evaluation for sensitivity and specificity of potential biomarkers

Immuno-reactive antigens will be upheld through a combined blood screening strategy to evaluate their sensitivity and specificity. The detection of monoclonal IgG antibodies for the evaluation of parasite-specific antigens/epitopes against malaria or schistosomiasis, and later to echinococcosis or African trypanosomiasis, will be carried out through ELISA-based assays or dipsticks using plasma samples of infected individuals from different endemic areas. Subsequently, the highly sensitive and specific biomarkers will be used to develop diagnostic kits for population use.

## Discussion

High-throughput biomarker screening platform has been established to identify suitable serological targets for diagnostics. This project focuses on four of the so called ‘diseases of the poor’: malaria (vivax and falciparum), schistosomiasis, echinococcosis and African sleeping sickness whose burden is enormous especially in African countries, with the initial target on malaria and schistosomiasis. The technology proves to be innovative in addressing major limitations of current ‘traditional’ diagnostic methods, such as parasitological or clinical methods that are neither simple nor convenient for population surveillance, and showed low sensitivity and specificity [14, 15]. Evaluation and validation tests of target candidates using a large repertoire of infected individual sera through antigen-based assays will guide the sensitivity and specificity, and also aid the prioritization of suitable biomarkers for the development of diagnostic kits. In addition, the project will evaluate and validate any new diagnostic through comparison with standard methods. For instance, in the case of *Schistosoma*, Kato-Katz stool examination will be used in comparison to new diagnostic, and microscopic examination of blood smears in comparison to new *Plasmodium* diagnostic*.*

Our expectations and future plans are to develop, evaluate, validate and optimize field deployable tests for each agent applicable to each region. More importantly, it is also imperative to seek regulatory approval and promote the use of products in endemic areas. However, exciting questions such as: how will the proposed diagnostics be affordable?, what will be the cost per test?, etc., have caught our attention and specific mechanisms need to be addressed.

## Additional file

Additional file 1: Multilingual abstract in the five official working languages of the United Nations. (PDF 554 kb)

## Abbreviations

ANDI: African Network for Drugs and Diagnostics Innovation
BCA: Bicinchoninic acid
CSA: Circulating *Schistosoma* antigen
DALY: Disability-adjusted life year
ELISA: Enzyme-linked immunosorbent assay
GPI: Glycosylphosphatidylinositol
HRP: Horseradish peroxidase
HTP: High-throughput
IgG: Immunoglobulin G
LB: Luria-Bertani
MSP: Merozoite surface protein
NDI: Network for drugs and diagnostics innovation
NIPD: National institute of parasitic diseases
NTDs: Neglected tropical diseases
ORF: Open reading frame
PCR: Polymerase chain reaction
RDT: Rapid diagnostic test
SDS-PAGE: Sodium dodecyl sulfate polyacrylamide gel electrophoresis
VSP: Variant surface protein
WGCF: Wheat germ cell-free
WHA: World health assembly
WHO: World health organization
