# Supplementary material for: Study roadmap for high-throughput development of easy to use and affordable biomarkers as diagnostics for tropical diseases: a focus on malaria and schistosomiasis
Source: Infect Dis Poverty. 2017 Oct 2;6:130. doi: 10.1186/s40249-017-0344-9 (PMC5623970; doi:10.1186/s40249-017-0344-9)
Supplement: Additional file 1: — Multilingual abstract in the five official working languages of the United Nations. (PDF 554 kb) [file 40249_2017_344_MOESM1_ESM.pdf]

Translation of the abstract into the five official working languages of the United Nations

خارطة طريق لدراسة تطوير إنتاجية عالية من السهل استخدامها وعلامات حيوية بأسعار معقولة كأدوات تشخيص للأمراض الاستوائية: التركيز على الملاريا وداء البلهارسيات

كوكوفي كاسيغن، تينغ تشانغ، شين بو تشن، بن شو، تشى شنغ دانغ، وانغ بينغ دنغ، إنيولا مايكل آبي، هاي مو شين، وى هو، تاكيل جريسو جويو، سولومان نواكا، جون هو تشن، شياو نونغ تشو

ملخص
خلفية: </bx>تستخدم التدخلات حالياً ضد 'الأمراض المعدية من الفقر'، التي تظل شديدة الوهن والقاتلة في معظم البلدان الموبوءة، وبخاصة الملاريا والبلهارسيا، وداء المشوكات ومرض النوم الأفريقي. ومع ذلك، فالقيود الرئيسية من الطرق "التقليدية" الحالية للتشخيص ليست بسيطة ولا ملائمة لمراقبة السكان، وأظهرت حساسية منخفضة وخصوصية. وهناك حاجة ماسة للحصول على تكنولوجيات جديدة من أجل استحداث أدوات كافية وموثوق بها. يهدف المشروع التعاوني بين "الشبكة الأفريقية" للأدوية وابتكار وسائل التشخيص والمؤسسات الشريكة في أفريقيا والصين إلى فحص المؤشرات الحيوية المصلية المناسبة للسبل التشخيصية ضد 'أمراض الفقراء' هذه.
الأساليب: تم فحص الأفراد المعرضين لطفيل محدد مقابل الأفراد الذين لم يتعرضوا له وجمعت الأمصال أو البول/البراز من خلال دراسات ضبط الحالة في الصين والدول الأفريقية. تم اختيار الجينات المستهدفة / إطارات القراءة المفتوحة، </ex> ثم سيتم استنساخها وإظهارها خالية من الخلايا، وتحديدها والكشف عن المناعية. وسيتم فحص المستضدات المستهدفة / المحددات المستضدية ومناظرتها مع المصل من الأفراد المعرضين أو غير المعرضين باستخدام منصة فحص المستضدات عالية الإنتاجية مع تقدم الدراسة. وسيتم تقييم خصوصية وحساسية المؤشرات الحيوية عالية المناعية أيضاً، وذلك باستخدام هاالإنزيم المرتبط بالفحص المناعي أو أدوات القياس.
المناقشة: وتكشف خارطة الطريق هذه بوضوح إجراءات التشغيل المتكاملة مع التركيز على الملاريا والبلهارسيا، لتحديد المؤشرات الحيوية المناسبة التي من شأنها أن تساعد في تحديد أولويات التشخيص لاستخدام السكان ومع ذلك، هناك حاجة إلى مواصلة التحقق من صحة أي تشخيص جديد من خلال المقارنة مع الأساليب القياسية في الاختبارات الميدانية القابلة للنشر لكل منطقة. توقعاتنا للمستقبل هي السعي للحصول على موافقة تنظيمية وتشجيع استخدام وسائل التشخيص في المناطق الموبوءة.

Translated from English version into Arabic by Free bird, through

TRANSLATORS
WITHOUT BORDERS

基于高通量技术的经济便捷型热带病诊断生物标志物研发路线：聚焦疟疾与血吸虫病

Kokouvi Kassegne, Ting Zhang, Shen-Bo Chen, Bin Xu, Zhi-Sheng Dang, Wang-Ping Deng, Eniola Michael Abe, Hai-Mo Shen, Wei Hu, Takele Geressu Guyo, Solomon Nwaka, Jun-Hu Chen, Xiao-Nong Zhou

摘要
引言：“贫困所致传染病”造成患者身体虚弱或致死，特别是疟疾、血吸虫病、包虫病和非洲昏睡病等。多数流行国家采用各种干预措施来防治这些疾病。然而，目前所使用的传统诊断方法在人群监测工作中使用不便，而且灵敏度和特异性较低。因此急需开发更可靠的诊断新技术。非洲药物与诊断创新网络及其合作机构正在进行一项国际合作研究，旨在筛选针对“贫困所致传染病”诊断过程所需的血清学生物标志物。
方法：对中国和非洲国家的病例对照研究中，通过检测挑选寄生虫特异性感染与未感染的个体，获取其血清或尿/粪便样本。选择靶基因及编码区，然后进行克隆和无细胞表达，定量并获取免疫表征。随后使用高通量抗原

筛选平台，用感染或未感染个体的血清来探测和筛选靶基因抗原表位。研究同时还使用酶联免疫吸附试验或免疫印迹法，来评估高免疫反应生物标志物的特异性和灵敏度。
讨论：本研究路线图以疟疾和血吸虫病为例，展示了寻找生物标志物及确定诊断工具的操作流程。然而对于不同的地区，本研究策略仍然需要与当地的标准检测方法进行比较来进一步验证新诊断技术的有效性。本研究期望相关诊断工具在未来能得到注册许可，并促进在热带病流行区的推广应用。

Translated from English version into Chinese by Jun-Hu Chen

Protocol d'Étude Pour le Développement à Haut Débit de Biomarqueurs Accessibles et Faciles à Utiliser Comme Agents Diagnostiques pour les Maladies Tropicales: Priorité pour la Malaria et la Schistosomiase

Kokouvi Kassegne, Ting Zhang, Shen-Bo Chen, Bin Xu, Zhi-Sheng Dang, Wang-Ping Deng, Eniola Michael Abe, Hai-Mo Shen, Wei Hu, Takele Geressu Guyo, Solomon Nwaka, Jun-Hu Chen, Xiao-Nong Zhou

Résumé

Contexte: Des interventions sont actuellement utilisées contre les maladies infectieuses dites 'de la pauvreté', notamment la malaria, la schistosomiase, l'échinococcose et la maladie du sommeil, qui restent très débilitantes et mortelles dans la plupart des pays endémiques. Toutefois, d'importantes limitations des méthodes 'traditionnelles' de diagnostic ne sont ni simples, ni commodes pour une surveillance appropriée de la population, et ont montré une faible sensibilité et spécificité. L'accès à de nouvelles technologies pour la mise au point des outils adéquats et fiables sont expressément nécessaires. Un projet de collaboration entre le Réseau Africain pour l'Innovation des Médicaments et Agents Diagnostiques (ANDI) et les institutions partenaires en Afrique et en Chine vise à identifier des biomarqueurs sérologiques appropriés pour le developpemt d'agents diagnostiques contre ces 'maladies des pauvres'.
Méthodes: Des individus exposés et non exposés aux parasites ont été dépistés et les échantillons de sérums et d'urine ou de selles ont été recueillis en Chine et dans des pays Africains à travers des études cas - témoins. Des gènes cibles/cadres de lecture ouverts (CLOs) ont été sélectionnés, suivi de l'expression acellulaire des protéines qui seront ensuite quantifiées et immuno-détectées. Les antigènes cibles/épitopes seront immuno-analysés avec des sérums de personnes exposées ou non à l'aide d'une plateforme de criblage à haut débit au fur et à mesure que l'étude progresse. La spécificité et la sensibilité des biomarqueurs fortement immunoréactifs seront aussi évaluées en utilisant les méthodes de dosage immunoenzymatique (ELISA) ou de bandelettes réactives.
Discussion: Cette feuille de route scientifique dévoile explicitement des procédures opérationnelles intégrées pour l'identification et le developpement d'agents diagnostiques apropriés, tout en donnant d'abord la priorité à la malaria et la schistosomiase. Toutefois, il est nécessaire de valider tout nouvel agent diagnostique en comparaison avec les procédés standard de diagnostic par des tests déployables sur le terrain pour chaque région. Nos attentes pour l'avenir sont d'obetnir l'approbation réglementaire et de promouvoir l'utilisation des agents diagnostiques dans les zones endémiques.

Translated from English version into French by Kokouvi Kassegne

Hoja de ruta para el desarrollo de de alto rendimiento de biomarcadores fо́ciles de usar y asequibles como diagnо́stico para las enfermedades tropicales: un enfoque en la malaria y la esquistosomiasis

Kokouvi Kassegne, Ting Zhang, Shen-Bo Chen, Bin Xu, Zhi-Sheng Dang, Wang-Ping Deng, Eniola Michael Abe, Hai-Mo Shen, Wei Hu, Takele Geressu Guyo, Solomon Nwaka, Jun-Hu Chen, Xiao-Nong Zhou

Resumen

Antecedentes: En la actualidad, se utilizan intervenciones contra «las enfermedades infecciosas de la pobreza», que siguen siendo altamente debilitantes y mortales en la mayoría de los países endémicos, especialmente para tratar la malaria, la esquistosomiasis, la equinococosis y la enfermedad del sueño africana. Sin embargo, lasimportantes limitaciones de los métodos «tradicionales» de diagnóstico actuales no son simples ni convenientes para la vigilancia de la población, y han demostrado tener una baja sensibilidad y especificidad. Claramente, es necesario teneracceso a tecnologías innovadoras para desarrollar herramientas adecuadas y fiables. Un proyecto de colaboración entre la Red Africana de Innovación en Farmacología y Diagnóstico e instituciones asociadas en África y China tiene como objetivo examinar biomarcadores serológicos adecuados para canales de diagnóstico contra estas «enfermedades de la pobreza».

Métodos: Se examinó a personas expuestas a determinados parásitos para compararlas con personas no expuestas, y se recolectaron muestras de suero u orina/heces a través de estudios de casos de control en China y en países africanos. Se seleccionaron genes objetivo/marcos de lectura abiertos, que luego serán clonados y expresados sin células, así como cuantificados e inmunodetectados. Los antígenos/epítopos objetivo se examinarán y seleccionarán a partir de suero de individuos expuestos o no expuestos usando una plataforma de detección de antígenos de alto rendimiento a medida que avance el estudio. También se evaluarán la especificidad y la sensibilidad de biomarcadores altamente inmunorreactivos, utilizando un ensayo de inmunoabsorción de unión enzimática o tiras reactivas.

Discusión: En esta hoja de ruta se exploran explícitamente los procedimientos operativos integrados centrados en la malaria y la esquistosomiasis, para la identificación de biomarcadores adecuados que ayuden a priorizar los diagnósticos para el uso de la población. Sin embargo, es necesario validar aún más cualquier nuevo diagnóstico mediante la comparación con métodos estándar en ensayos que puedan implementarse en campo para cada región. Nuestras expectativas para el futuro son procurar la aprobación regulatoria y promover el uso del diagnostico en áreas endémicas.

Translated from English version into Russian by Liudmila Tomanek (nee Volynets) , through

TRANSLATORS
WITHOUT BORDERS

Hoja de ruta para el desarrollo de de alto rendimiento de biomarcadores fáciles de usar y asequibles como diagnóstico para las enfermedades tropicales: un enfoque en la malaria y la esquistosomiasis

Kokouvi Kassegne, Ting Zhang, Shen-Bo Chen, Bin Xu, Zhi-Sheng Dang, Wang-Ping Deng, Eniola Michael Abe, Hai-Mo Shen, Wei Hu, Takele Geressu Guyo, Solomon Nwaka, Jun-Hu Chen, Xiao-Nong Zhou

Resumen

Antecedentes: En la actualidad, se utilizan intervenciones contra «las enfermedades infecciosas de la pobreza», que siguen siendo altamente debilitantes y mortales en la mayoría de los países endémicos, especialmente para tratar la malaria, la esquistosomiasis, la equinococosis y la enfermedad del sueño africana. Sin embargo, lasimportantes limitaciones de los métodos «tradicionales» de diagnóstico actuales no son simples ni convenientes para la vigilancia de

la población, y han demostrado tener una baja sensibilidad y especificidad. Claramente, es necesario teneracceso a tecnologías innovadoras para desarrollar herramientas adecuadas y fiables. Un proyecto de colaboración entre la Red Africana de Innovación en Farmacología y Diagnóstico e instituciones asociadas en África y China tiene como objetivo examinar biomarcadores serológicos adecuados para canales de diagnóstico contra estas «enfermedades de la pobreza».

Métodos: Se examinó a personas expuestas a determinados parásitos para compararlas con personas no expuestas, y se recolectaron muestras de suero u orina/heces a través de estudios de casos de control en China y en países africanos. Se seleccionaron genes objetivo/marcos de lectura abiertos, que luego serán clonados y expresados sin células, así como cuantificados e inmunodetectados. Los antígenos/epítopos objetivo se examinarán y seleccionarán a partir de suero de individuos expuestos o no expuestos usando una plataforma de detección de antígenos de alto rendimiento a medida que avance el estudio. También se evaluarán la especificidad y la sensibilidad de biomarcadores altamente inmunorreactivos, utilizando un ensayo de inmunoabsorción de unión enzimática o tiras reactivas.

Discusión: En esta hoja de ruta se exploran explícitamente los procedimientos operativos integrados centrados en la malaria y la esquistosomiasis, para la identificación de biomarcadores adecuados que ayuden a priorizar los diagnósticos para el uso de la población. Sin embargo, es necesario validar aún más cualquier nuevo diagnóstico mediante la comparación con métodos estándar en ensayos que puedan implementarse en campo para cada región. Nuestras expectativas para el futuro son procurar la aprobación regulatoria y promover el uso del diagnostico en áreas endémicas.

Translated from English version into Spanish by María Emilia Meini, through

TRANSLATORS
WITHOUT BORDERS
